# Frequency of *ABCA4* mutations in 278 Spanish controls: an insight into the prevalence of autosomal recessive Stargardt disease

**DOI:** 10.1136/bjo.2008.148155

**Published:** 2008-10-31

**Authors:** R Riveiro-Alvarez, J Aguirre-Lamban, M Angel Lopez-Martinez, M Jose Trujillo-Tiebas, D Cantalapiedra, E Vallespin, A Avila-Fernandez, C Ramos, C Ayuso

**Affiliations:** 1 Fundación Jimenez Diaz, Genetics Department, Madrid, Spain; 2 Centro de Investigacion Biomedica en Red de Enfermedades Raras (CIBERER), ISCIII, Madrid, Spain

## Abstract

**Aim:**

To determine the carrier frequency of *ABCA4* mutations in order to achieve an insight into the prevalence of autosomal recessive Stargardt disease (arSTGD) in the Spanish population.

**Methods:**

arSTGD patients (n = 133) were analysed using ABCR400 microarray and sequencing. Control subjects were analysed by two different strategies: 200 individuals were screened for the p.Arg1129Leu mutation by denaturing-HPLC and sequencing; 78 individuals were tested for variants with the microarray and sequencing.

**Results:**

For the first strategy in control subjects, the p.Arg1129Leu variant was found in two heterozygous individuals, which would mean a carrier frequency for any variant of ∼6.0% and a calculated arSTGD prevalence of 1:1000. For the second strategy, carrier frequency was 6.4% and therefore an estimated prevalence of the disease of 1:870.

**Conclusion:**

Calculated prevalence of arSTGD based on the *ABCA4* carrier frequency could be considerably higher than previous estimation. This discrepancy between observed (genotypic) and estimated (phenotypic) prevalence could be due to the existence of non-pathological or low penetrance alleles, which may result in late-onset arSTGD or may be implicated in age-related macular degeneration. This situation should be regarded with especial care when genetic counselling is given and further follow-up of these patients should be recommended.

Stargardt disease (STGD1, MIM #248200) is the most common hereditary macular dystrophy affecting children, with a prevalence of approximately 1:10 000 and a carrier frequency of ∼2%.^1^ It is characterised by central visual loss, atrophy of the retinal pigment epithelium (RPE) that resembles a “beaten-bronze” appearance, and the distribution of orange-yellow flecks around the macula and midperiphery of the retina.^2^ STGD is predominantly inherited as an autosomal recessive trait, although an autosomal dominant form has been also described.^3^ Bi-allelic mutations in *ABCA4* are found in most patients with autosomal recessive STGD (arSTGD)^4^ and in some patients with autosomal recessive retinitis pigmentosa (arRP),^5^ autosomal recessive cone-rod dystrophy (arCRD)^6^ and age-related macular degeneration (AMD).^7^

Up to now, approximately 500 disease-causing mutations have been identified in *ABCA4*. The mutation spectrum ranges from single base substitutions to deletions of several exons or uniparental disomies,^8^ ^9^ although the majority of reported changes are missense mutations (http://www.hgdm.cf.ac.uk/ac/index.php). In addition, heterozygote carrier frequency is particularly high in the general population.^10^ Previous mutational analyses performed in Spanish arSTGD patients led to the identification of a prevalent disease-associated allele, the p.Arg1129Leu variant.^11^

Because of the relatively large size of this gene, containing 50 exons, the molecular scanning of *ABCA4* is particularly labour-intensive. The ABCR400 chip (Asper Biotech, Tartu, Estonia) has become a reliable and rapid mutation detection tool and, as previously described, was >98% effective in screening for disease-associated alleles.^10^ However, because of the high frequency of rare mutations reported in this gene, additional mutational scanning should be carried out by denaturing-HPLC (dHPLC).^12^ This results a more effective screening tool for *ABCA4* variants than previous methods such as double gradient-denaturing gradient gel electrophoresis (DG-DGGE).^13^ This technique is especially applicable for those *ABCA4* patients for whom the chip found one or no mutations. Obtained results from both techniques should be confirmed by direct sequencing, which is still considered the “gold standard” method for mutation detection, and enables the identification from 66–80% of the *ABCA4* disease-associated alleles.^14^ ^15^

The aim of this study was to determine the frequency of *ABCA4* mutations in control individuals. According to these mutational findings and allelic frequencies calculations such as the Hardy–Weinberg equilibrium, we aimed to achieve an insight into the prevalence of arSTGD in the Spanish population. To overcome this challenge we designed a case–control study that combined several high-throughput and cost-effective screening tools such as ABCR400 genotyping microarray, dHPLC and direct sequencing.

## Patients and methods

A case–control study was designed. Methods are described as follows.

### Recruitment of patients

Thorough clinical ophthalmic and electrophysiological examinations were performed in arSTGD patients, including comprehensive ophthalmological and family history, funduscopic examination after pupillary dilation, static perimetry, best corrected visual acuity, colour vision testing and fluorescein angiography. Electrophysiological assessment included full-field electroretinogram (ERG) according to standards of the International Society for Clinical Electrophysiology of Vision (ISCEV).^16^ ^17^

### Ascertainment of controls

Healthy control individuals were recruited from anonymous blood donors from the blood service of the Fundacion Jimenez Diaz Hospital, Madrid, Spain. Subjects had previously given informed consent where their nationality, age and sex were stated. Recent immigrants were excluded from the study, thus ensuring a homogeneous genetic background.

### Molecular approach in arSTGD patients

Data from 133 arSTGD patients were analysed using the ABCR400 genotyping microarray;^10^ results were confirmed by direct sequencing.

### Molecular approach in control population

**Figure 1:**
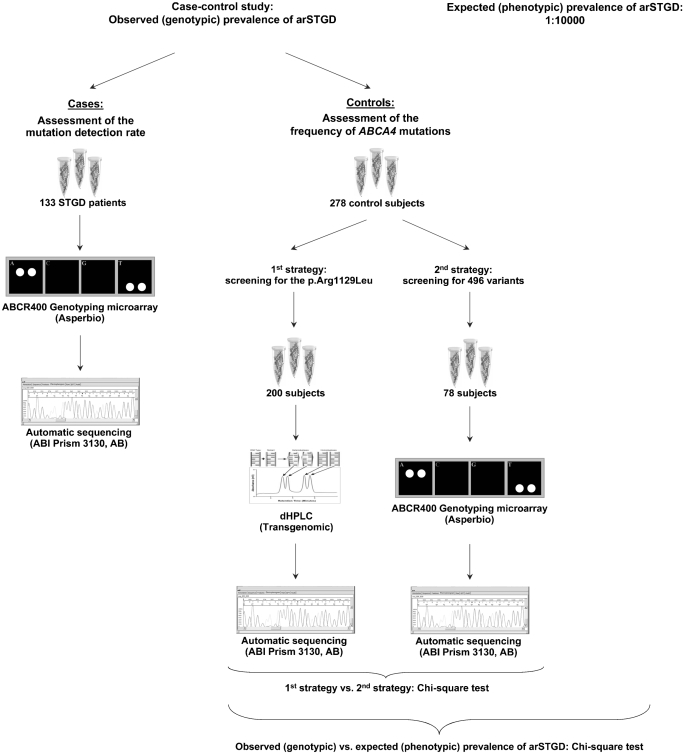
Algorithm representing the case–control study: two different molecular approaches were performed; results were compared by statistical analysis. ar, autosomal recessive; dHPLC, denaturing-HPLC; STGD, Stargardt disease.

Control individuals were analysed following to two different strategies in order to compare both results and to further determine whether there were significant differences. For both strategies, allelic frequencies were estimated by the Hardy–Weinberg equilibrium (*p*^2^+2*pq*+*q*^2^ = 1) and 95% CI was calculated for obtained carrier frequencies; statistical data were analysed by the chi-square test (fig 1).

#### First strategy

As previously described,^11^ the frequency of the p.Arg1129Leu allele is notably high in Spanish patients. Therefore, 200 subjects (400 chromosomes) from a control population were tested for this variant by cost-effective dHPLC technique. Those samples presenting an abnormal chromatographic pattern were sequenced. The carrier frequency for any *ABCA4* variant was then extrapolated.

#### Second strategy

In a different subset of 78 subjects (156 chromosomes), the *ABCA4* carrier frequency was determined by screening samples on the ABCR400 microarray; results were confirmed by direct sequencing.

### Molecular methods

#### DNA extraction

Peripheral blood samples were taken and genomic DNA was extracted using an automated DNA extractor (BioRobot EZ1, QIAGEN, Hilden, Germany).

### Genotyping microarray

arSTGD patients were analysed for variants on the ABCR400 microarray (www.asperbio.com), as described elsewhere.^10^ The 50 exons of the *ABCA4* gene, including the intron–exon junctions, were amplified by PCR primers previously described,^18^ in order to confirm the results obtained from the microarray.

### Direct sequencing

Sequencing reactions were performed using the four dye terminator cycle sequencing ready reaction kit (dRhodamine DNA Sequencing Kit; Applied Biosystems, Foster City, California, USA). Sequence products were purified through fine columns (Sephadex G-501, Princetown Separations, Adelphia, New Jersey, USA) and resolved in an ABI Prism 3100 (Applied Biosystems).

### dHPLC

dHPLC sample screening was performed on a DNA fragment analysis system (WAVE, Transgenomic, Omaha, Nebraska, USA), as described elsewhere.^12^

## Results

### Cases

Seventy-six of the 133 Spanish arSTGD patients have been previously reported on by our group.^11^ Nevertheless, they have been included in the present study in order to achieve a more accurate mutation detection rate for the genotyping microarray, as it is currently updated with novel disease-associated alleles.

**Figure 2:**
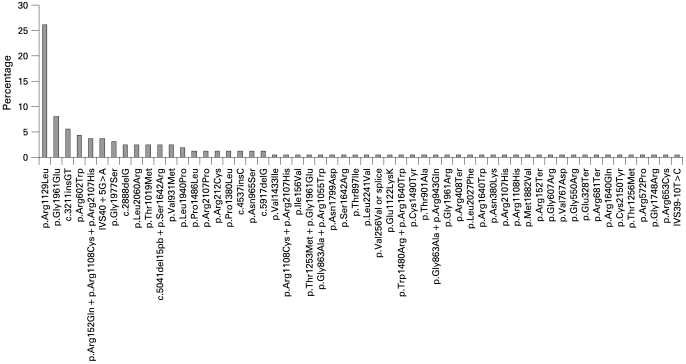
The spectrum of *ABCA4* disease-associated alleles identified in Spanish Stargardt disease (STGD) patients and relative frequencies. Of these, the prevalent p.Arg1129Leu variant represented the 26% of the mutant alleles.

Of the 266 arSTGD chromosomes studied, mutations were identified in 161, resulting in a detection rate for the genotyping microarray of 60.5%: two mutant alleles were found in 64/133 patients (48.1%), whereas in 33/133 cases (24.8%) only one allele could be identified. A total of 56 different sequence variants related to the disease were identified, including missense (42), nonsense (four) frameshift (five) and splicing (five) mutations. Furthermore, combinations of variants acting in *cis* (complex alleles; six double alleles, one triple allele) were also found. Of this spectrum of substitutions, the p.Arg1129Leu (c.3386G>T) allele accounted for 26% of the disease-associated alleles (fig 2). Considering the percentage represented by this variant in patients (26%) and the obtained mutation detection rate (60.5%), this change would represent the 15.7% of all the potential arSTGD associated alleles.

### Controls

#### Analysis of 400 chromosomes

Because of its high frequency in the group of patients with arSTGD, we determined the presence of the p.Arg1129Leu mutation in 200 control individuals from Spain. By use of dHPLC, this allele was found in two heterozygous individuals (allelic frequency = 2/400 = 0.005). Next, we extrapolated the carrier frequency for any *ABCA4* variant (absolute mutant frequency; *q* value), considering the current frequency of the p.Arg1129Leu allele in arSTGD patients (15.7%), resulting in *q* = 0.032. Given these allelic frequencies, we applied the Hardy–Weinberg equilibrium, obtaining an estimated heterozygous carrier frequency (2*pq* value) of 5.99% with 95% CI 1.35 to 10.63. Therefore, the arSTGD prevalence would be 1:1000.

#### Analysis of 156 chromosomes

Following the screening on the ABCR400 chip, ten heterozygous individuals were identified. Interestingly, this fraction includes only missense disease-associated alleles. No additional variants were found in the remaining controls, but common polymorphisms (table 1). Therefore, carrier frequency was 6.4% (ten mutant alleles out of 156) with 95% CI 1.62 to 11.18. By applying the Hardy–Weinberg equilibrium, an estimated prevalence of the disease of 1:870 was obtained.

**Table 1:** *ABCA4* sequence variants identified in Spanish control population

	Mutant alleles					
	Nucleotide change	Amino acid change	Number of cases	Number of alleles	Frequency (%)	Homozygous individuals
Mutations*	c.661G>A	p.Gly221Arg	1	1	0.64	None
	c.1140T>A	p.Asn380Lys	1	1	0.64	None
	c.2588G>C	p.Gly863Ala	1	1	0.64	None
	c.3113C>T	p.Ala1038Val	1	1	0.64	None
	c.3899G>A	p.Arg1300Gln	1	1	0.64	None
	c.5882G>A	p.Gly1961Glu	1	1	0.64	None
	c.5908C>T	p.Leu1970Phe	1	1	0.64	None
	c.6148G>C	p.Val2050Leu	1	1	0.64	None
	c.6529G>A	p.Asp2177Asn	2	2	1.28	None
Total			10			
Polymorphisms†	c.466A>G	p.Ile156Val	5	5	3.2	None
	c.635G>A	p.Arg212His	5	6	3.84	1
	c.1268A>G	p.His423Arg	43	48	30.7	5
	c.1269C>T	p.His423His	2	2	1.28	None
	IVS10+5delG		34	36	23	2
	c.2828G>A	p.Arg943Gln	1	1	0.64	None
	c.4203C>A	p.Pro1401Pro	3	3	1.9	None
	IVS33+48C>T		59	75	48	16
	c.5603A>T	p.Asn1868Ile	4	4	2.5	None
	c.5682G>C	p.Leu1894Leu	29	35	22.4	6
	c.5814A>G	p.Leu1938Leu	27	33	21.1	6
	c.5843 C>T	p.Pro1948Leu	9	10	6.4	1
	c.5844A>G	p.Pro1948Pro	27	32	20.5	5
	c.6069C>T	p.Ile2023Ile	11	12	7.7	1
	c.6249C>T	p.Ile2083Ile	12	14	8.9	2
	c.6285T>C	p.Asp2095Asp	24	26	16.6	2
	c.6764G>T	p.Ser2255Ile	12	13	8.3	1

*A total of 15 mutant alleles were detected.

†Polymorphisms identified in control individuals: eight out of 16 were synonymous codon variants. In addition, the decision criteria for being non-pathogenic changes were supported by the existence of unaffected individuals harbouring homozygous variants.

### Statistical analysis

#### Differences between genotyping strategies in controls

Calculated prevalence values resulting from both genotyping strategies (p.Arg1129Leu screening, prevalence: 1:1000 vs. microarray screening, prevalence: 1:870) were compared using the chi-square test, but this analysis did not reveal a significant difference (α = 0.05).

#### Differences between prevalence estimation in controls vs arSTGD prevalence

The chi-square test (α = 0.05) was significant when comparing previous prevalence (1:10 000) with the present results.

#### Differences of the frequency of the p.Arg1129Leu allele between patients and controls

In addition, the frequency of p.Arg1129Leu variant was tested between in arSTGD patients and controls. The chi-square test (α = 0.05) showed significant differences among both groups, confirming more association in the patient group.

## Discussion

Rare diseases are those considered to be less frequent than 1:3000. This classification comprises the heterogeneous group of inherited retinal dystrophies, which includes both central (macular) or peripheral retinopathies. Up to now, arSTGD was thought to be the most common juvenile macular dystrophy, but still considered within the group of rare diseases. Current prevalence of the disease has been established in one affected individual per 10 000 live births,^1^ thus indicating a carrier frequency of approximately 2%.

To evaluate the arSTGD prevalence further, we proposed two different strategies for determining the carrier frequency of *ABCA4* disease-associated alleles in a Spanish control population. In one strategy, the arSTGD prevalence was calculated over the frequency of the most common allele (p.Arg1129Leu) in 400 ethnically matched control chromosomes. In the other, 78 control subjects were directly screened for variants and the subsequent prevalence of the disease was assessed. These molecular approaches were based on the mutation detection rate of the high-throughput ABCR400 microarray, which we previously tested^11^ and confirmed in Spanish arSTGD patients (present study). Jaakson *et al*^10^ reported that the array is theoretically able to detect about 56% of the disease-associated alleles in populations of European origin. In our set of samples, including 76 previously analysed arSTGD patients, 161 potential disease-associated alleles were identified, yielding a detection rate of 60.5% by the ABCR400 chip. Considering these results, mutation detection might only be increased by additional molecular scanning performed either by dHPLC or direct sequencing. Both techniques are the most suitable tools for the identification of novel *ABCA4* variants not yet contained in the chip.

As Valverde *et al* previously reported,^11^ the patterns of the disease-associated alleles were significantly different from those described at other European populations. In our Spanish arSTGD patients, the most frequent mutation was the missense p.Arg1129Leu variant, accounting for 26% of the disease-associated alleles (fig 2), that is approximately one out of about four (3.84) of the arSTGD alleles. Given this high frequency for a specific allele, the control population was tested for this variant. We were able to identify that the presence of this sequence change was significantly higher in patients than in controls (chi-square test; α = 0.05). The p.Arg1129Leu allele was found in two heterozygous individuals out of 278 (two heterozygous individuals out of 200 screened by dHPLC; zero individuals out of 78 screened by the ABCR400 chip), yielding an allelic frequency of 0.36%; this supports the notion of a pathogenic change as this variant is less frequent than 1%. Moreover, the biochemical characterisation of a recombinant ABCR protein with the p.Arg1129Leu mutation revealed a substantial reduction in both expression and ATP-binding activity.^19^ In our 133 STGD families, seven were homozygous for the p.Arg1129Leu allele (five of them have been described previously).^11^ In addition, haplotype analysis with markers flanking the *ABCA4* gene (TEL-*D1S435*-*D1S2804*-*ABCA4*-*D1S236*-CEN) showed co-segregation of the disease within these families. Moreover, mutation segregation was observed among unaffected members of these cohorts (results available on request).

The frequency of this mutation in the control population would mean an estimated carrier frequency for any *ABCA4* variant of 5.99% (95% CI 1.35 to 10.63). In consequence, the estimated arSTGD prevalence would be 1:1000, which is ten times higher than current 1:10 000 value. Furthermore, this result was confirmed by a second control population screening performed by the ABCR400 microarray. This analysis resulted in a carrier frequency of 6.4% (95% CI 1.62 to 11.18) and therefore, an estimated prevalence of the disease of 1:870, which is more that 11.5 times greater than 1:10 000. When comparing both strategies, statistical analyses did not show significant differences. Nevertheless, the tests were significant when comparing our results with previous prevalence of arSTGD.

Different techniques, with distinct mutation detection rates, have been used to assess the heterozygote carrier frequency. Nevertheless, the comparison between them seems reasonable since all results have been confirmed by direct sequencing, which is still considered the gold standard method. However, these estimations of prevalence should be regarded with certain caution, as they depend highly on one assumption (the p.Arg1129Leu frequency in arSTGD patients should be proportional to the p.Arg1129Leu frequency in controls) that is never entirely fulfilled.

Whereas the precise prevalence of arSTGD is still not known, we have observed a discrepancy between the observed (genotypic) values (1:1000; 1:870) and the estimated (phenotypic) (1:10 000) prevalence of the disease. This difference could be due to the existence of combinations of different alleles that are not pathological or, at least, do not result in an early-onset phenotype (low penetrance alleles). Therefore, this approach might be used to assess the amount of pathogenicity attributable to each *ABCA4* allele. For example, if we get a high arSTGD prevalence caused by a concrete allele, this would mean that this variant is a polymorphism or a “mild allele” rather than a pathogenic change. Indeed, it has been suggested that the homozygous mild c.2588G>C allele does not cause arSTGD.^20^ If the prevalence of the disease was calculated over the frequency of this common variant it would result in a prevalence of 1:58 in European populations, which reinforces the hypothesis of being a mild allele and only being pathogenic when associated with another severe to moderate disease-causing allele.

By contrast, if the relative allelic frequency for one change is low, that change could be classified as a disease-causing allele. Moreover, this last hypothesis would be reinforced by the scarcity of a concrete allele together with having a frameshift or splicing effect, and possibly associated with a more severe phenotype, such as cone-rod dystrophy or retinitis pigmentosa. In fact, the c.2888delG variant has been described as the most frequent allele in Spanish cone-rod dystrophy patients, whereas it has not been associated with the arSTGD phenotype.^21^

Regarding to the genetic diagnosis and counselling of arSTGD patients, a few points should be considered. (1) High-throughput techniques are able to detect a wider spectrum of mutations in *ABCA4* than before, achieving higher mutation detection rates. (2) Accurate clinical diagnosis and thorough ophthalmic examination of inherited retinopathies are now possible, thus providing a more comprehensive management of the disease and offering the possibility of molecular analysis. (3) Previous studies have found that 5–12% of the general population carry disease-associated *ABCA4* alleles.^7^ ^10^ ^18^ ^20^ In this study, we have identified significant percentages of carriers, with values of about 20% (table 2).

**Table 2:** Comparative analysis of carrier frequencies of *ABCA4* disease-associated alleles in control individuals from different populations

Screening tools	Confirmation method	No. of control chromosomes	No. of mutated control chromosomes	Frequency (alleles) (%)	Frequency (individuals) (%)	Reference	Population
DGGE+dHPLC+SSCP	Direct sequencing	440	27	6.1	12.3	Rivera *et al* (2000)^18^	Germany
SSCP+HA (heteroduplex analyses)	Direct sequencing	440				Allikmets *et al* (1997)^7^	USA
ASO (c.2588G>C)		622	9	1.4	2.9	Maugeri *et al* (1999)^20^	The Netherlands
ASO (c.2588G>C+c.2828G>A)		308	2	0.6	1.3	Maugeri *et al* (1999)^20^	The Netherlands
ASO (c.2828G>A)		308	9	2.9	5.8	Maugeri *et al* (1999)^20^	The Netherlands
ABCR400 array	SSCP+direct sequencing	192	9	4.7	9.4	Jaakson *et al* (2003)^10^	USA
dHPLC+ABCR400 array (p.Arg1129Leu)	Direct sequencing	556	2	0.36	0.72	Present study	Spain
ABCR400 array	Direct sequencing	156	15	(9.6)	(19.2)	Present study	Spain

ASO, allele-specific nucleotide; DGGE, denaturing gradient gel electrophoresis; dHPLC, denaturing-HPLC; HA, heteroduplex analyses; SSCP, single-strand conformation polymorphism.

Moreover, if we consider that this value has been obtained through screening on the ABCR400 chip (mutation detection rate = 60.5%) and the high frequency of rare mutations reported in this gene, this percentage could be even higher. These molecular findings might suggest a higher prevalence of arSTGD and, in consequence, a need for revision of the present value. However, this estimated prevalence does not correlate with the expected prevalence of the disease and questions the pathogenic significance of some mutations. The combination of these alleles with low penetrance should be regarded especially carefully when genetic counselling is given and further follow-up of these patients should be recommended in order to establish an accurate genotype–phenotype correlation. (4) The finding of a higher carrier frequency should be carefully considered when dealing with retinal dystrophies resembling any of the phenotypes attributable to *ABCA4*: arSTGD, arCRD or arRP. In addition, as being carrier of an *ABCA4* disease-associated allele should give an increased risk for AMD,^7^ healthy parents or siblings of arSTGD patients should receive an appropriate eye care when diagnosed, especially before becoming elderly.

For those populations where the spectrum of *ABCA4* mutations is known and considering that allelic frequencies differ from one country to another, the use of a cost-effective strategy similar to the one described in this study, based on carrier frequency screenings, might be recommended to assess the estimated (genotypic) prevalence of arSTGD. Moreover, this type of approach can also be applied to other non-retinal diseases where the mutational spectrum is well-known or the evidence of a prevalent disease-associated allele has been demonstrated.
